# Restoration of Tear Secretion in a Murine Dry Eye Model by Oral Administration of Palmitoleic Acid

**DOI:** 10.3390/nu9040364

**Published:** 2017-04-05

**Authors:** Shigeru Nakamura, Yuki Kimura, Daisuke Mori, Toshihiro Imada, Yusuke Izuta, Michiko Shibuya, Hisayo Sakaguchi, Erina Oonishi, Naoko Okada, Kenji Matsumoto, Kazuo Tsubota

**Affiliations:** 1 Department of Ophthalmology, Keio University School of Medicine, Tokyo 160-8582, Japan; fd3sima@gmail.com (T.I.); qqwp4kyq.izt@gmail.com (Y.I.); rmshibuya@gmail.com (M.S.); sono.hisayo2@gmail.com (H.S.); e.oonishi@a5.keio.jp (E.O.); snoopy-naoko@mvh.biglobe.ne.jp (N.O.); tsubota@z3.keio.jp (K.T.); 2 Gifu Shellac Manufacturing Co., Ltd., Gifu 500-8618, Japan; gsm.rd@gifushellac.co.jp (Y.K.); gsm.rd2@gifushellac.co.jp (D.M.); 3 Department of Allergy and Clinical Immunology, National Research Institute for Child Health & Development, Tokyo 157-8535, Japan; matsumoto-k@ncchd.go.jp

**Keywords:** fatty acid, ophthalmology, dry eye

## Abstract

Sea buckthorn (*Hippophae rhamnoides)*–derived products have traditionally been used as food and medicinal ingredients in Eastern countries. The purpose of this study was to investigate the effect of oral intake of sea buckthorn oil products on tear secretion using a murine dry eye model. Orally administered sea buckthorn pulp oil (not seed oil) restored aqueous tear secretion to its normal value under a dry eye condition. Palmitoleate (C16:1), a fatty acid present in sea buckthorn pulp oil, preserved tear secretion and suppressed inflammatory cytokines in the lacrimal gland to the same extent as that by pulp oil. These results suggest that an oral intake of sea buckthorn pulp oil has a potency to preserve tear secretion capacity in the dry eye state and palmitoleate, its main constituent fatty acid, is an active component of the oil. This effect may enable a potent diet-based treatment for the prevention of dry eye.

## 1. Introduction

Sea buckthorn (*Hippophae rhamnoides*) is a deciduous splinter shrub plant of the Elaeagnaceae family with yellow or orange berries, which is widely grown in the central and northern areas of Eurasia including Russia, China, Mongolia, France, The Netherlands, Finland, Sweden, and Norway [1]. It has traditionally been used for nutritional and medicinal purposes in various countries. A large number of studies have shown a wide range of biological activities and potential health benefits involving all parts of the plant [2,3,4]. The pulp of its berries contains abundant vitamins, minerals, amino acids, and polyphenolic compounds, which have been reported to contribute to human health benefits [5]. Oils extracted from the pulp or seed have traditionally been used for treating mucosal disorders such as dermatitis [6]. In addition, dried or fresh leaves are prepared for nutritious herbal tea as they are rich in nutraceutical components [7].

Aqueous tear fluid is secreted from the lacrimal gland (LG), and constantly flows over the ocular surface to create a moistened, properly controlled environment for the conjunctiva and avascular cornea. Dry eye syndrome is characterized by impairment of the status of the tear film, which causes ocular discomfort accompanied by visual impairment. It is becoming a public health problem that reduces the daily quality of life [8]. The incidence of dry eye syndrome is markedly increasing in industrialized countries due to the expanding usage of information technology devices [9]. Temporal aqueous tear supplementation therapy, the frequent application of artificial tear eye drops, has long been adopted for the basic management of dry eye symptoms. 

A recent study showed that the dietary intake of sea buckthorn oil attenuated tear hyperosmolality and subjective symptoms in patients with dry eye syndrome [10]. This study suggested that sea buckthorn oil products have the potential to improve the secretory function of tears in dry eyes. However, a suitable source and the active components of this oil have yet to be examined in detail. Sea buckthorn oil contains a high content of various polyunsaturated and saturated fatty acids [11,12]. These fatty acids have been recognized as essential nutrients for energy sources, vital structural components and important cell signaling molecules with multiple biological effects [13]. In the present study, we assessed the usefulness of sea buckthorn oil products on dry eyes. Specifically, we evaluated the effects of the constituent polyunsaturated and saturated fatty acids found in sea buckthorn oil on tear secretion capacity and studied their anti-inflammatory effects on lacrimal glands using two murine dry eye models.

## 2. Materials and Methods

### 2.1. Oil Products and Fatty Acids

Sea buckthorn was collected from Germany. Sea buckthorn pulp oil (PO), and seed oil (SO) were extracted by crushing and centrifugation (Sanddorn GbR, Herzberg, Germany).

### 2.2. Chemicals

The α-linolenate, linolate, stearate, palmitate, and oleate were purchased from Tokyo Chemical Industry Co., Ltd. (Tokyo, Japan). Palmitoleate (POA) was purchased from, Cayman Chemical Co., (Michigan, MI, USA).

### 2.3. Animals

Eight-week-old female Sprague-Dawley rats (*n* = 6–8 in each experiment) and seven-week-old female C57BL/6N mice (*n* = 5–7 in each experiment) were purchased from Charles River (Yokohama, Japan). They were quarantined and acclimatized before the experiments for one week, under standard conditions, as follows: room temperature of 23 ± 2 °C, relative humidity of 60% ± 10%, alternating 12 h light-dark cycle (8 a.m.–8 p.m.), and water and food available ad libitum.

All animal experiments were approved by the Animal Care and Use Committee of Keio University (Approval No. 12111–1), and all procedures were performed in accordance with the Association of Research and Vision in Ophthalmology (ARVO) statement for the Use of Animals in Ophthalmic and Vision Research.

### 2.4. Murine Dry Eye Models

We used a mouse stress-induced dry eye model to screen oil products from sea-buckthorn and fatty acids components [14] and a rat blink-suppressed dry eye model to simulate effect on dry eye in patient whose etiology is associated with excess staring at a computer display [15].

### 2.5. Mouse Stress-Induced Dry Eye Model

Mice were physically restrained in a 50 mL plastic conical tube and subjected to a stream of air directed at their heads, at a rate of 0.5~1.0 m/s for four hours. They were placed individually in cages, with water and food available ad libitum for the remaining time. Each material was homogeneously suspended in distilled water by vigorous vortex mixing and orally administered once a day to mice in doses of 0.5 and 2.5 mL/kg, prior to stress exposure. Distilled water served as the vehicle control. The dosage of fatty acids corresponded to the content of PO described in Table 1. The treatment duration was one day in screening of oil products and fatty acids, and 10 consecutive days in measurement of serum fatty acid and lacrimal gland cytokine concentration.

### 2.6. Rat Blink-Suppressed Dry Eye Model

A series of treatments was performed under dry conditions, with a room temperature of 23 ± 2 °C, relative humidity of 25% ± 5%, and a constant air flow of 2 to 4 m/s produced by an 18-cm-diameter electric fan. Sustained suppression of blinking in the rat was achieved by keeping the rat stationary on a swing made of plastic piping (30 × 50 mm), suspended 60 cm above the floor. After 4 h on the swing, the rats were returned to their cages for 30 min for food and water, and again placed on the swing for 3.5 h. They were individually placed in cages with water and food ad libitum for the remaining 16 h. This series of treatments was repeated for 10 days. Sea buckthorn pulp oil or olive oil (2.5 mL/kg) was homogeneously suspended in 1 mL of distilled water by vigorous vortex mixing and administered orally to rats once a day for 10 consecutive days, prior to the swing procedure. Distilled water served as the vehicle control.

### 2.7. Measurement of Aqueous Tear Secretion

In the mouse study, tear secretion was measured using a phenol red thread (Zone-Quick; Showa Yakuhin Kako Co., Ltd., Tokyo, Japan). The threads were placed on the medial canthus for 15 s. The length of the moistened area from the edge was then measured to within 0.5 mm. Tear secretion was measured before the oil product administration and after the end of stress exposure. The data are represented as percentages of the pre-administration values. For the mouse tear secretion measurement, five to seven mice were used from each group.

In the rat study, modified Schirmer test was used under topical anesthesia induced with a 0.4% oxybuprocaine hydrochloride solution (Santen Pharmaceutical, Osaka, Japan). Tear secretion was measured before the oil product administration of every treatment day and 18 h after the last time the rat was kept stationary on a swing on day 10 (day 11). After 3 min of anesthesia, a phenol red thread (Zone-Quick; Syowa Yakuhin Kako, Ltd., Tokyo, Japan) was placed on the temporal side of the lower eyelid margin for one minute. The length of the moistened area from the edge was then measured to within 1 mm. For the rat tear secretion measurement, six rats were used from each group.

### 2.8. Measurement of Fatty Acids in Oil Products and Mouse Serum

The lipid extract was methylated with trimethylsulfonium hydroxide (TMSH, Tokyo Chemical Industry Co., Ltd.) and heptadecanoic acid (Wako Pure Chemical Industries Ltd., Osaka, Japan) as an internal standard and analyzed as methyl esters using gas chromatography (GC, GC-17A, Shimadzu Co., Kyoto, Japan). CP-Sil 88 capillary column (100 m × 0.25 mm inner diameter × 0.2 μm film thickness; Agilent Technologies, Inc., Folsom, CA, USA) was used. Gas N2, split 1:100, injection: 1 μL. The temperature program was as follows: initial at 80 °C with a 1 min hold; ramp: 4 °C/min to 220 °C, hold: 220 °C for 5 min; ramp: 220 °C at 4 °C/min to 230 °C, hold: 230 °C for 19 min. The injector was set at 230 °C and detector was set at 300 °C.

The oil products (5 mg) were dissolved in 940 μL of tert-Butyl methyl ether (TBME; Wako Pure Chemical Industries, Ltd., Osaka, Japan) which contained internal standard and 60 μL of TMSH (0.2 mol/L in methanol) and applied to GC analysis. The measurements of fatty acids in the tested materials are shown in Table 1.

In the mouse study, 2.5 mL/kg of sea-buckthorn pulp oil was orally administered for once a day for 10 consecutive days. Two hours after the final administration, mouse blood was collected from the tail vein. Serum was separated by centrifugation at 15,000× *g* for 10 min, and lipid extract was extracted with 100 μL ×3 of TBME which contained the internal standard. The organic phases were combined and evaporated in a vacuum centrifuge. The dried lipid extract was dissolved in 90 μL of TBME and 10 μL of TMSH (0.2 mol/L in methanol) and subjected to GC analysis. For the mouse serum fatty acid measurement, five mice were used from each group.

### 2.9. Lacrimal Gland Cytokine Measurement

Two and a half mL/kg of sea buckthorn pulp oil and 0.75 mg/kg POA were repeatedly orally administered once a day for 10 consecutive days in a mouse stress-induced dry eye model. The lacrimal gland was excised and homogenized in 0.2 mL of ice-cold PBS with a Polytron homogenizer (Kinematica AG Inc., Lucerne, Switzerland). The homogenate was diluted to 3 mg/mL protein concentration. Cytokine concentrations were determined using a mouse-specific Milliplex cytokine/chemokine kit according to the manufacturer’s instruction. Values were calculated based on a standard curve constructed for the assay. For the mouse lacrimal gland cytokine measurement, four (PO and POA) or five (vehicle) mice were used.

### 2.10. Statistical Analysis

Student’s *t*-test was used to compare the two groups and Dunnett’s test was used for multiple comparisons. Differences between the measured variables were considered significant if the resultant *p*-value was 0.05 or less.

## 3. Results

### 3.1. Fatty Acid Compositions of Sea Buckthorn Oil Products and Olive Oil

The content and composition of the fatty acids in sea buckthorn oil has been reported to vary based on the origin of its production [11]. First, we analyzed the fatty acid composition of PO, SO, and olive oil used in this study (Table 1). The measured fatty acid species were referenced from a previous analytical paper [11].

For the PO, palmitate, palmitoleate (POA) and oleate were approximately 30 mg/100 mg and the others were less than 3 mg/100 mg. For the SO, oleate, linolate, and α-linoleate were approximately 20 mg/100 mg. The concentrations of palmitate, palmitoleate, and oleate were 11.3, 7.5, and 2.0 mg/100 mg, respectively. Pulp oil contained higher contents of palmitate, oleate, and POA (1.5- to three-fold) and lower contents of lionate and α-linolenate (approximately 1/10) compared with SO. In contrast, olive oil contained 78.4 mg/100 mg of oleic acid and less than 10 mg/100 mg of the other five fatty acids.

### 3.2. The Effects of Sea Buckthorn Oil Products on Tear Secretion

**Figure 1:**
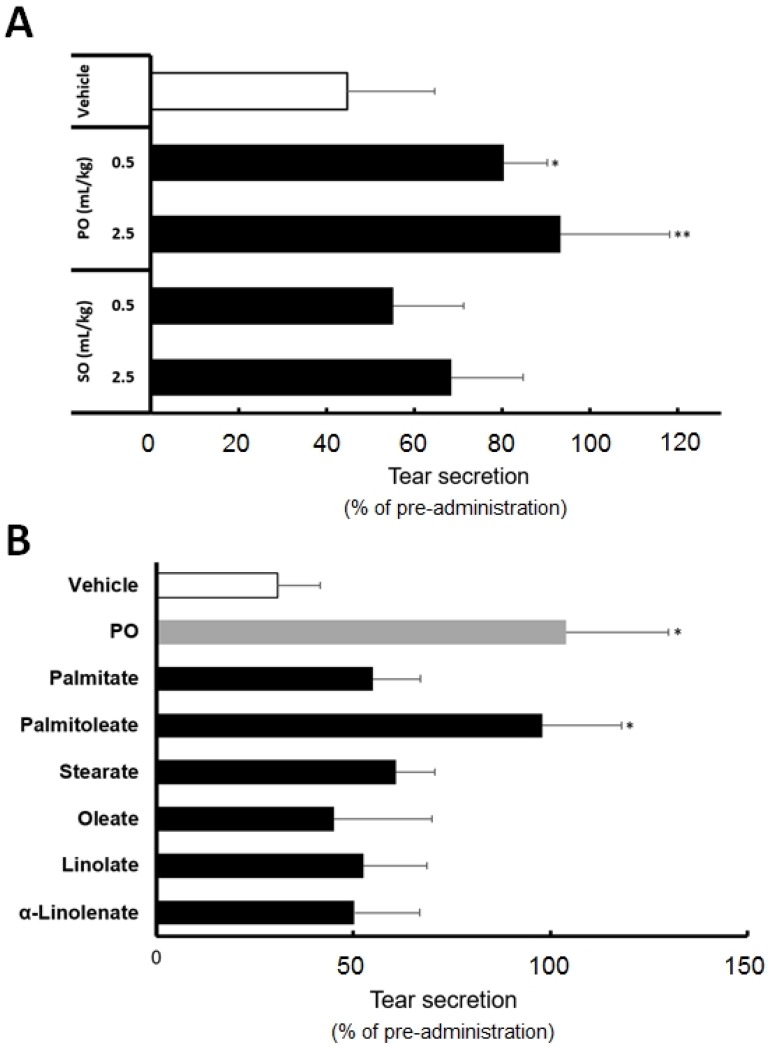
Effect of sea buckthorn oil products and their constituent fatty acids on tear secretion in mouse stress-induced dry eye model. (**A**) Effect of sea buckthorn pulp oil (PO) and seed oil (SO); (**B**) Effect of constituent fatty acids in sea buckthorn oil products. The data are represented as percentages of the pre-administration values. The dosage of fatty acids is corresponded to the content in PO described in Table 1. All data represent the mean ± SD of five to seven mice. * *p* < 0.05, ** *p* < 0.01 versus the vehicle.

To identify the effective oil products from sea buckthorn, we evaluated the effects of PO and SO fatty acid compositions on the tear secretion. Figure 1A shows a decrease in the tear secretion after stress exposure following different SO and PO administrations. Tear secretion in the mouse dry eye model was lowered approximately by 45% in the vehicle group compared to the pre-stress exposure values. The oral administration of 0.5 and 2.5 mL/kg PO reduced the impairment in tear secretion by 93% and 80%, respectively, compared to the pre-PO administration values prior to the stress exposure. This suppression was significantly higher than that exhibited by the vehicle (*p* < 0.05 at 0.5 mL/kg and *p* < 0.01 at 2.5 mL/kg).

The oral administration of 0.5 and 2.5 mL/kg SO lowered the tear secretion by approximately 55% and 68%, respectively, compared to the pre-SO administration values prior to the stress exposure. No significant changes were observed with SO for the dosage administered, compared to the vehicle.

### 3.3. Palmitoleate Is an Active Component of Sea Buckthorn Pulp Oil

Figure 1B shows the effects of six PO-constituting fatty acids on tear secretion. We compared their effects at a dosage corresponding to our analytical results of PO (Table 1). Among the six fatty acids, palmitate, POA, oleate, α-linolenate, linolate, and stearate, POA preserved tear secretion to the same level as PO. The values for PO and POA were 104% and 98%, respectively, compared to the pre-POA administration values prior to the stress exposure. Significant tear preservation was observed with POA and PO, compared to the vehicle (*p* < 0.05). Tear secretion was reduced after the administration of the other four fatty acids by 50% to 60%, compared to the pre-administration values prior to the stress exposure. No significant changes were observed with the other fatty acids compared to the vehicle. 

### 3.4. Orally Administrated Sea Buckthorn Pulp Oil Changed Serum Fatty Acid Composition

**Figure 2:**
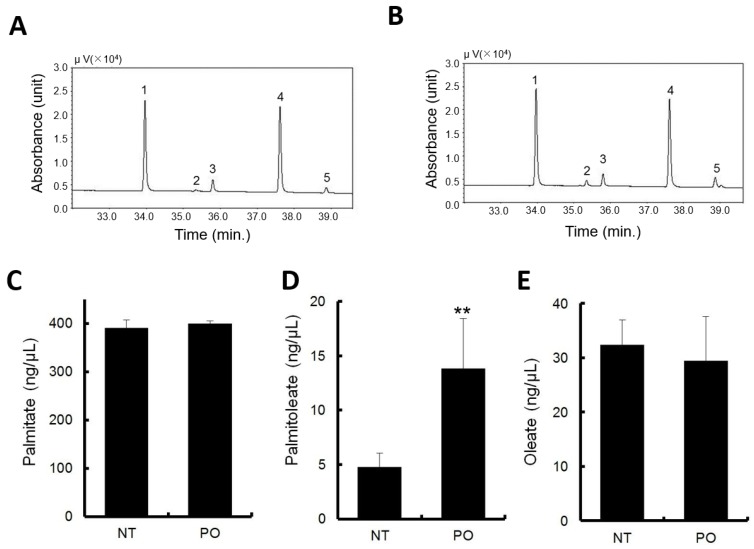
Sea buckthorn pulp oil administration increased serum palmitoleate. Gas chromatograms of non-treatment (**A**) and PO treatment (**B**) sera. Peak identification: 1, palmitate; 2, palmitoleate (POA); 3, internal standard; 4, stearate; 5, oleate; Serum concentration of (**C**) palmitate, (**D**) POA, (**E**) oleate. All data represent the mean ± SD of five mice. ** *p* < 0.01, versus the vehicle.

The major constituent fatty acids of PO are endogenously present in normal mouse serum as metabolites of fatty acid metabolism. In the vehicle administration, fatty acid concentrations were approximately 400 ng/μL for PA, 40 ng/μL for oleate, and POA was approximately 5 ng/μL, being the lowest among the fatty acids measured (Figure 2D–F). 

PO administration elevated the POA levels approximately three times the level in normal serum (Figure 2D). No significant changes were observed with PA and POA, compared to normal serum (Figure 2C,E).

Typical gas chromatograms of non-treatment and PO treatment are shown in Figure 2A,B, respectively.

### 3.5. Changes of Cytokine and Chemokine Expression in the LG after Repeated Treatment with Sea Buckthorn Pulp Oil or Palmitoleate

Changes in cytokines and chemokines in the lacrimal gland were evaluated for 10 days with repeated treatment with PO (2.5 mL/kg) or POA (0.75 mg/kg), at a dosage corresponding to the content in PO.

**Figure 3:**
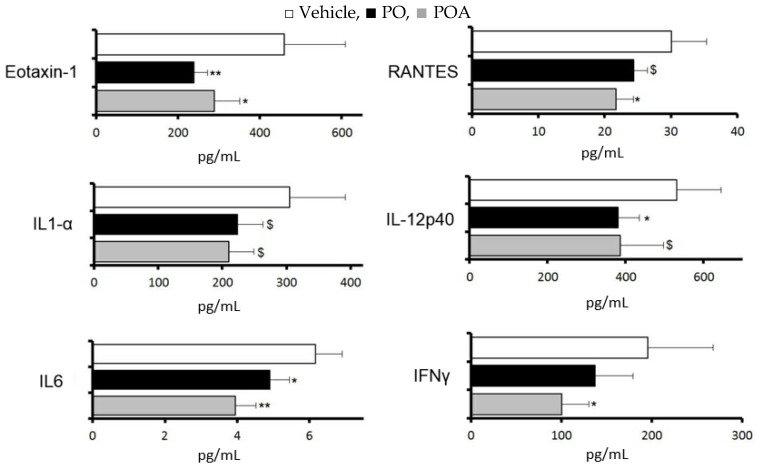
Sea buckthorn pulp oil and palmitoleate suppressed inflammatory cytokines and chemokines in the lacrimal gland in a similar manner. The dosage of POA corresponded to the content in PO (Table). □ Vehicle, ■ pulp oil (PO), 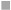 palmitoleate (POA). All data represent the mean ± SD of four to five mice. ^$^ *p* < 0.1, * *p* < 0.05, ** *p* < 0.01 versus the vehicle.

With PO, significant suppressions were observed in eotaxin-1, interleukin 12p40, and interleukin 6, and a tendency towards suppression was observed in RANTES and interleukin 1 alpha (*p* < 0.1). With POA, significant suppression was observed in eotaxin-1, RANTES, and interleukin 6, and a tendency towards suppression was observed in interleukin 12p40 and interleukin 1 alpha (*p* < 0.1, Figure 3).

### 3.6. Sea Buckthorn Pulp Oil, Not Olive Oil, Restores Tear Secretion in a Rat Blink-Suppressed Dry Eye Model

The effect of PO on tear secretion under dry eye conditions was compared with that of olive oil, whose health benefits as a natural ingredient have been well reported [16].

**Figure 4:**
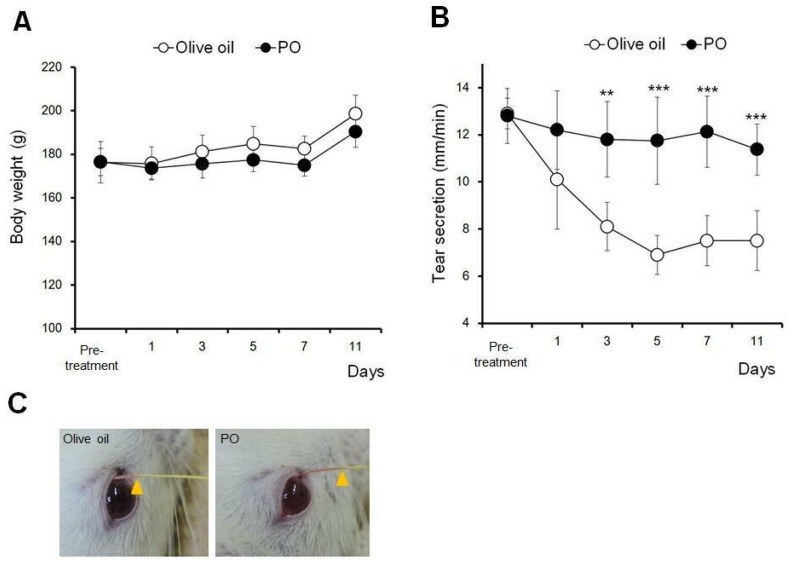
Sea buckthorn pulp oil, and not olive oil, restored tear secretion in a rat blink-suppressed dry eye model. (**A**) Changes in body weight (*n* = 6 rats); (**B**) Changes in tear secretion. (*n* = 6 rats). All data represent the mean ± SD. ** *p* < 0.01. *** *p* < 0.005 versus olive oil; (**C**) Typical tear secretion patterns measured by a cotton thread. The arrow shows the wetted length by caused tear secretion. Sea buckthorn pulp oil (PO).

Figure 4 shows the effects of 10 days of repeated oral administration of 2.5 mL/kg PO on body weight and tear secretion. No significant difference was observed in the body weight among PO and olive oil (Figure 4A). 

In the olive oil administration group, the tear secretion gradually decreased and plateaued, at approximately 50% of the pre-treatment values, between days 5 and 11. Repeated oral administration of PO had no further effect on the tear secretion during the dry eye treatment (Figure 4B). Significant differences were observed between days 3 and 11, compared to the olive oil group (*p* < 0.01 at day 3 and *p* < 0.005 at days 5 to 11). Figure 4C shows the representative photographs of the tear secretion patterns measured using the modified Schirmer test.

## 4. Discussion

Sea buckthorn PO showed a more potent effect to preserve tear secretion than SO. Our quantitative data on the fatty acid composition of sea buckthorn oils showed that there are stark differences between the pulp and seed. Pulp oil contains higher contents of palmitate, oleate, and POA (1.5- to three-fold) and lower contents of lionate and alpha-linolenate (approximately 1/10) compared to SO. In addition, animal data showed that high levels of palmitate and oleate, and not of POA, were present in mouse serum in normal conditions, and that the administration of PO significantly increased the serum POA level. Together with the result that POA and not palmitate corrected the decrease in tear secretion in the dry eye model, it is suggested that the constituent POA of PO is an active component for resolving tear secretion dysfunction in dry eye conditions.

POA is a 16-carbon, omega-7, monounsaturated fatty acid that is biosynthesized from palmitate by fatty acid desaturase in adipose tissue [17]. POA is not only an intermediate of the fatty acid metabolic cascade, but it also exhibits a role in various physiological functions [18]. This fatty acid acts as a major signaling lipid hormone that strongly stimulates muscle insulin action which leads to improving glucose metabolism and suppressing hepatosteatosis with anti-inflammatory effects [18]. Supplementation with POA has been shown to attenuate liver or vascular inflammation caused by metabolic stress [19,20,21]. Inflammation is potentially involved in the pathogenesis of dry eye [22,23,24]. Consistent with the effects on the tear secretion capacity, the changes in cytokine and chemokine expression in the lacrimal gland after repeated treatment with PO were similar to those with POA. Interleukin-12p40 (IL-12p40), the bioactive cytokine of IL-12, is a pivotal inflammatory mediator in barrier tissues, and induces T lymphocytes to express Interferon-γ (IFN-γ) [25]. IFN-γ and interleukin-1α (IL-1α) are pleiotropic cytokines involved in the immune response and are potent chemoattractants for eosinophils and lymphocytes (Eotaxin-1) in the course of the inflammatory processes [26,27]. Interleukin-6 (IL-6) is a cytokine produced by immune and non-immune cells, and it acts as a mediator for several acute-phase inflammatory responses [28]. RANTES is a CC chemokine targeting eosinophils, T cells, and monocytes, and is produced by a variety of cell types including platelets and fibroblasts. IL-1α, IL-6, IL-12, IFN-γ, and RANTES have been documented to be increased in the tears of dry eye patients [29,30]. Eotaxin, IFN-γ, and RANTES have been shown to be involved in lacrimal gland destruction [25,31,32]. Therefore, our results suggest that POA exhibits the potential to preserve the tear secretion capacity in dry eye conditions by suppressing lacrimal gland inflammation. Further investigations should address the mechanisms underlying the relationship between the anti-inflammatory effect of POA and lacrimal gland secretory function.

A Mediterranean diet has been identified as a specifically healthy diet pattern with favorable effects on various health outcomes [16]. Higher levels of consumption of olive oil are considered the main characteristics of this diet. In the present study, to estimate the potency of effectiveness of PO as an intervention to prevent dry eye, the effect of PO on the preservation of tear secretion was compared with olive oil using rat blink-suppressed dry eye model. The dry eye model used in this study was created to simulate the effect of the excessive use of digital devices by repeatedly exposing rats to a daily stressful condition—persistent strain by swinging in combination with exposure to an evaporation-promoting environment. Consistent with the findings of the epidemiological study on VDT workers, this rat model showed a persistent decrease in tear secretion that was dependent on the strain strength [15]. Contrary to expectations, repeated treatment with PO, not olive oil, significantly restored tear secretion in the rat blink-suppressed dry eye model that mimics the situation of office workers (Figure 4). Our analytical results of oil products (Table 1) showed that the fatty acid composition of olive oil differs from that of PO because of its high oleic acid (78.4 mg/100 g in this study) and low saturated and unsaturated fatty acid content, including POA (0.6 mg/100 g in this study) contents, compared to PO. This superior effect of SO compared to olive oil on aqueous tear secretion in dry eye conditions was possibly due to the higher content of POA. Together with the variety of health benefits of sea buckthorn based on human and animal studies, the present study revealed that the dietary intake of PO as a natural ingredient not only ameliorates dry eye but also shows health benefits similar to olive oil.

In conclusion, among sea buckthorn plant parts, the pulp oil is a potent candidate for dry eye treatment through the suppression of inflammation of the tear secretion organ, the lacrimal gland. Omega-7 monounsaturated fatty POA, a characteristic fatty acid in PO, was identified as an active agent. Further clinical studies involving the dietary intake of POA and focused on information technology–associated dry eye patients will help clarify the efficacy of sea buckthorn and establish new therapeutic interventions for this disease.

## Figures and Tables

**Table 1:** Fatty acid composition of sea-buckthorn oils and olive oil.

Fatty Acid	Sea-Buckthorn Oil		Olive Oil
	PO	SO	
Palmitate C16:0	30.0	11.3	9.7
Palmitoleate C16:1	30.1	7.5	0.6
Stearate C18	0.6	2.0	3.1
Oleate C18:1	28.6	18.5	78.4
Linolate C18:2	2.8	22.8	6.9
α-Linoleate C18:3	1.0	21.8	0.6

(mg/100 mg).

## Abbreviations

PO	Sea buckthorn pulp oil
SO	Sea buckthorn seed oil
POA	Palmitoleate
